# Phytochemistry, pharmacology, toxicology and detoxification of *Polygonum multiflorum* Thunb.: a comprehensive review

**DOI:** 10.3389/fphar.2024.1427019

**Published:** 2024-06-17

**Authors:** Jiawen Qian, Chenhang Feng, Ziyang Wu, Yuanmei Yang, Xiangfu Gao, Lingyan Zhu, Yang Liu, Yuancheng Gao

**Affiliations:** ^1^ Department of Nephrology, The First Affiliated Hospital of Zhejiang Chinese Medical University (Zhejiang Provincial Hospital of Chinese Medicine), Hangzhou, China; ^2^ The Third Affiliated Clinical Medical College, Zhejiang Chinese Medical University, Hangzhou, China; ^3^ School of Pharmacy, Fudan University, Shanghai, China; ^4^ Institute of Chinese Materia Medica, Shanghai University of Traditional Chinese Medicine, Shanghai, China; ^5^ Shaanxi Academy of Traditional Chinese Medicine, Xi’an, China

**Keywords:** Polygonum multiflorum Thunb., toxicology, chemical components, processing, traditonal Chinese medicine

## Abstract

**Background:**

Polygonum multiflorum Thunb. (PM), a kind of perennial plant, belongs to the genus *Polygonum* of the family polygonaceae.The dry root of PM (also called Heshouwu), is a traditional Chinese medicine, which has a series of functions and is widely used in clinic for hair lossing, aging, and insomnia. While, PM also has some toxicity, its clinical drug safety has been concerned. In this paper, the chemical components, toxic mechanisms and detoxification strategies of PM were reviewed in order to provide evidence for its clinical application.

**Materials and methods:**

We conducted a systematic review of published literature of PM, including English and Chinese databases, such as PubMed, Web of Science, CNKI, and Wanfang.

**Results:**

PM contains a variety of chemical compounds, including stilbenes, quinones, flavonoids, phospholipids, and has many pharmacological activities such as anti-aging, wound healing, antioxidant, and anti-inflammatory properties. The PE has certain therapeutic effect, and it has certain toxicity like hepatotoxicity, nephrotoxicity, and embryotoxicity at the same time, but.these toxic effects could be effectively reduced by processing and compatibility.

**Conclusion:**

It is necessary to further explore the pharmacological and toxicological mechanisms of the main active compounds of PE.This article provides scientific basis for the safe clinical application of PM.

## 1 Introduction

*Polygonum multiflorum* Thunb. (PM), also known as He Shou Wu (HSW), is a perennial vine of the Polygonaceae family. First documented in “Kai Bao Ben Cao,” HSW has been a staple in traditional Chinese medicine for centuries, treating a range of ailments including sores, age-related conditions, and anemia. Over 100 chemical constituents have been identified in PM, with variations in toxicity attributed to differences in these components. Both water and alcohol extracts of raw PM and processed ones have been studied, revealing distinct toxicological profiles. The primary toxicity of PM is hepatotoxicity, with mechanisms involving intrinsic and idiosyncratic factors. Traditional Chinese Medicine (TCM) employs processing and compatibility techniques to reduce toxicity and enhance therapeutic effects, which are practical for daily use and deserve further research. This review aims to elucidate the traditional uses, chemical composition, toxicology, and detoxification methods of PM, providing a foundation for future research and therapeutic development.

## 2 Traditional application of PM

**FIGURE 1:**
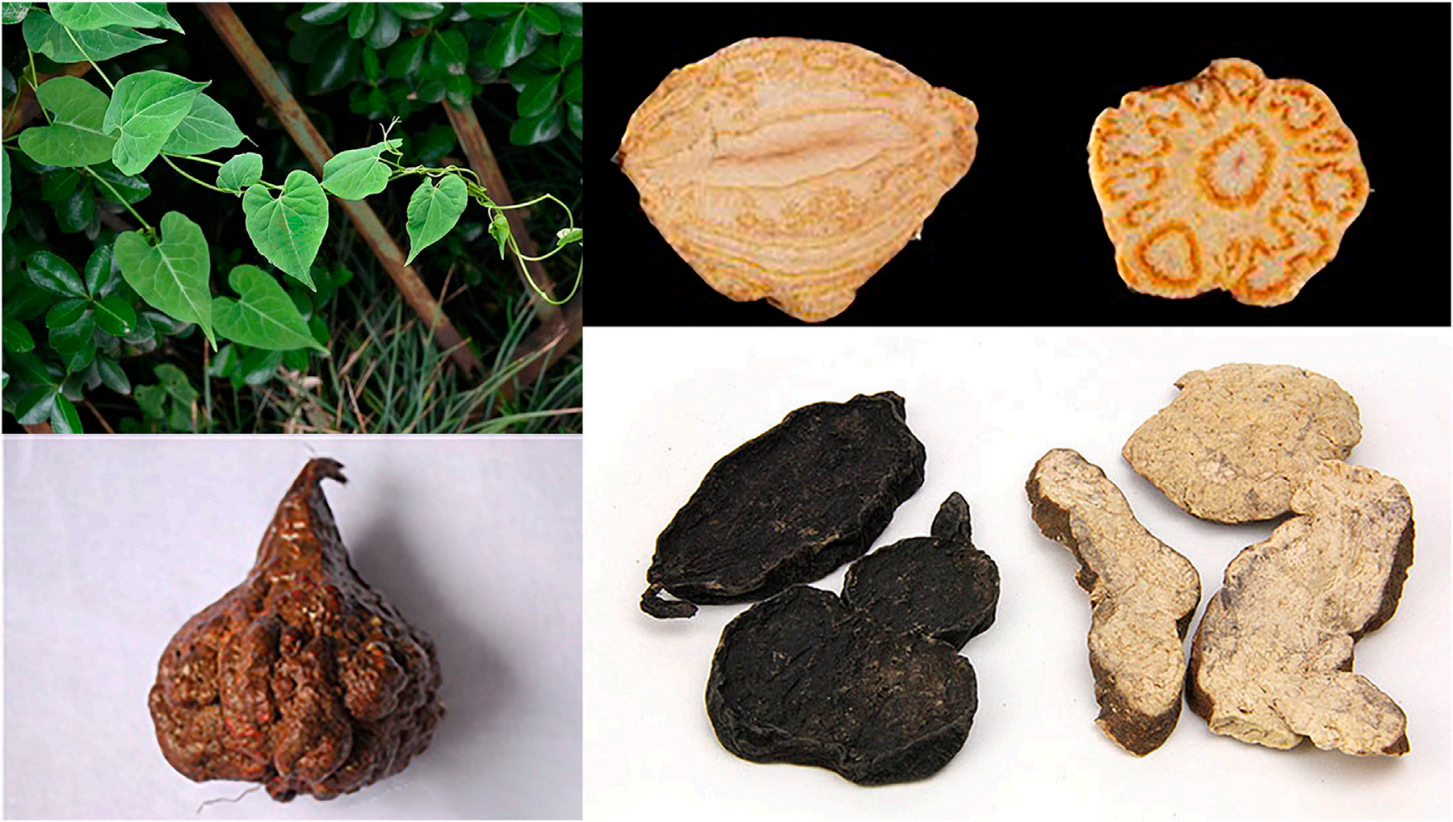
*Polygonum multiflorum* Thunb.: the plant, the medical section, the slices before and after processing.

“He Shou Wu” has been an official entry in the Chinese Pharmacopoeia since the Tang dynasty, over 1200 years ago. It was first recorded in “Kai Bao Ben Cao,” with pharmacological effects of anti-sores and anti-aging, treating anaemia and *postpartum* diseases. “Compendium of Materia Medica” stated that PM can treat the pain in the loins and knee, “Ben Cao Xin Bian,” “Ben Cao Qiu Zhen” recorded that PM is used to treat headache, hemorrhoids, cutaneous pruritus, and immune related disease. “Zhong Hua Yao Dian” also stated that PM can be used to treat constipation and hyperlipidemia (Figure 1).

PM is often used in conjunction with other herbs in formulations that address age-related conditions. Processed PM is usually used in age-related diseases. “Shi Bu Zhai Yi Shu” mentioned a formula named “Shou Wu Yan Shou Pill,” which can be used to treat hypertension and delaying the process of aging. There is another formula named “Qi Bao Mei Ran Pill,” which was stated in “Ji Shan Tang Fang,” it is used to treat hair losing. While raw PM is mainly used for treatment of *postpartum* fever, such as the formula of “He Ren Decoction” recorded in “Jing Yue Quan Shu”.

In summary, PM is extensively used to nourish the liver and kidneys, nourish blood, and dispel wind, exhibiting a wide range of pharmacological effects that differ between its raw and processed forms. Processed PM is predominantly used for liver and kidney yin deficiency, including conditions like premature graying, weakness of the waist and knees, muscle and bone pain, limb numbness, tinnitus, neurasthenia, hyperlipidemia, and spermatozoa issues. It is also used to nourish blood, commonly treating anemia and dizziness. Raw PM is mostly used in dispel wind. Raw PM is mostly used in treatment of long-term malaria, long-term dysentery, chronic hepatitis, scrofula and hemorrhoids (Supplementary Table S1).

Clinical trials have validated these pharmacological effects. For instance, one study found that the PM group showed the most significant increase in black hair, with higher levels of total melanin, α-MSH, MC1R, and TYR—key targets in PM’s use for hair graying. Chemical constituents other than TSG may contribute to the hair color regulation activity of PMR (Han et al., 2015). Another trial showed that the effect of PM extract on AD is superior to western medicine. The total effective rate of 93.33% in the PM extract treatment group was better than 73.33% in the Chinese herb control group and 68.97% in the western medicine control group (*p* < 0.01) (Chen et al., 2010). Additionally, Yadong Fan et al., 2021 investigated the anti-inflammatory activity of Tongmai Yangxin pill, a formula including PM, in treatment of coronary heart disease, and results showed that TMYX treatment showed reduced levels of apolipoprotein B, endothelin 1, nuclear factor κB (NF-κB) and homocysteine in CHD patients, suggesting the formula’s effectiveness in attenuating macrophage foam cell formation and its anti-inflammatory activity through modulation of the ESR1 and NF-κB signaling pathways.

## 3 The chemical components of PM

The chemical components of PM varies between its water and alcohol extracts. The chief toxic compositions in water extract are stilbenes, while in alcohol extract are quinones. More than 103 components are isolated and characterized in PM, including flavonoids, phospholipids, quinones, stilbenes, etc. Stilbenes and quinones are the main characteristic components in PM, with cases of hepatotoxicity reported.

### 3.1 The effective ingredients are generally extracted from raw/processed PM using water and alcohol

While some studies suggest that the water extract is less toxic than the alcohol extract, this remains a topic of debate. Water extract of PM treated groups showed significant inhibitions in CYP2E1 enzymatic activities and mRNA expressions (Li et al., 2015). However, another study compared the toxicity of water extract and alcohol extract of PM, and the result indicated that alcohol extract had much stronger hepatotoxicity than water extract, the content of emodin-8-O-β-D-glucopyranoside, physcion-8-O-β-D-glucopyranoside, emodin and physcion was significantly higher in alcohol extract than in water extract, while the human hepatocytes extraction showed that 2,3,5,4′-tetrahydroxystilbene-2-O-β-D-glucopyranoside, emodin-8-O-β-D-glucopyranoside, physcion-8-O-β-D-glucopyranoside, emodin and physcion had interaction with human hepatocytes (Lv et al., 2015). Yang Xuehuan et al. showed a different result, they proved that the toxicity of water extract was greater than that of alcohol extract. Based on metabolomics technology, they found that the liver toxicity of alcohol extract and water extract of PM was mainly caused by one carbon unit metabolism, arachidonic acid metabolism and glycerophospholipid metabolism (Wang et al., 2021). Main toxic components and toxic mechanisms of water extract (Table 1) and alcohol extract (Table 2) are as follows.

**TABLE 1:** Toxic components and toxic mechanisms of water extract of PM.

Water extract	
toxic components	Toxic mechanisms
stilbene glycoside, emodin 8-O-β-D glucoside, emodin and emodin methyl ether	Inhibit the mRNA expression of various CYP450 enzymes in human hepatocytes, resulting in liver metabolic dysfunction and liver injury
cis-stilbene glycoside	Inhibit PPAR-γ pathway to induce idiotypic liver injury
emodin	Inhibit the function of UGTs and MRP2 transporters to induce liver injury
emodin methyl ether	The liver metabolites inhibit UGT1A1 enzyme
trans-stilbene glycoside	Metabolizing through UGTs mediated phase II pathway, inhibition of UGTs can lead to dysfunction of the metabolism, inducing liver injury

**TABLE 2:** Toxic components and toxic mechanisms of alcohol extract of PM.

Alcohol extract	
toxic components	Toxic mechanisms
aloe emodin	reduce the survival rate of L02 cells and causes S phase block of hepatocytes
chrysophanol	Inhibit bile salt export pump and multidrug resistance-associated protein 2 to accumulate endogenous BA in the liver, may be related to P450 1A2, P450 2B6 and P450 3A4 in hepatocytes
tetrahydroxy stilbene-O-(galloyl)-hex	Unknown, lead to apoptosis of rat hepatocytes

Additional studies have explored other extracts of PM, scholars showed that ethyl acetate (EA) extract had close association with the idiosyncratic hepatotoxicity of PM. Co-treatment with non-toxic dose of lipopolysaccharide (LPS) and EA extract could result in evident liver injury, indicated by the significant elevation of plasma alanine aminotransferase (ALT) and aspartate aminotransferase (AST) activities, as well as obvious liver histologic damage (Li et al., 2016). Another study assessed in zebrafish embryos showed that the toxicity of 70% EtOH extract is considerably higher than that of other extracts (Yang et al., 2018).

In conclusion, both raw and processed PM pose risks to liver health, though the extent of toxicity varies among the different extracts. As a result, although the hepatotoxicity of processed product is reduced, caution should be taken in its clinical use.

### 3.2 The chemical components of PM are reported including flavonoids, phospholipids, quinones and stilbenes

PM is known for its diverse phytochemical composition, including flavonoids, phospholipids, quinones, and stilbenes. Notably, stilbenes and quinones stand out as key components due to their pharmacological properties and associated hepatotoxic risks, with compounds such as 2,3,5,4′-tetrahydroxystilbene-2-O-β-D-glucopyranoside and emodin dianthrones being particularly significant.

#### 3.2.1 Stilbenes

Stilbenes, significant non-flavonoid phytochemicals with a polyphenolic structure, are prevalent in mosses and various plants. Stilbenes can be used as medicine for their anti-oxidant, anti-proliferation and anti-inflammatory properties. However, they may impair UDP-glucuronosyltransferases (UGTs), potentially leading to drug-induced liver injury (DILI).

**FIGURE 2:**
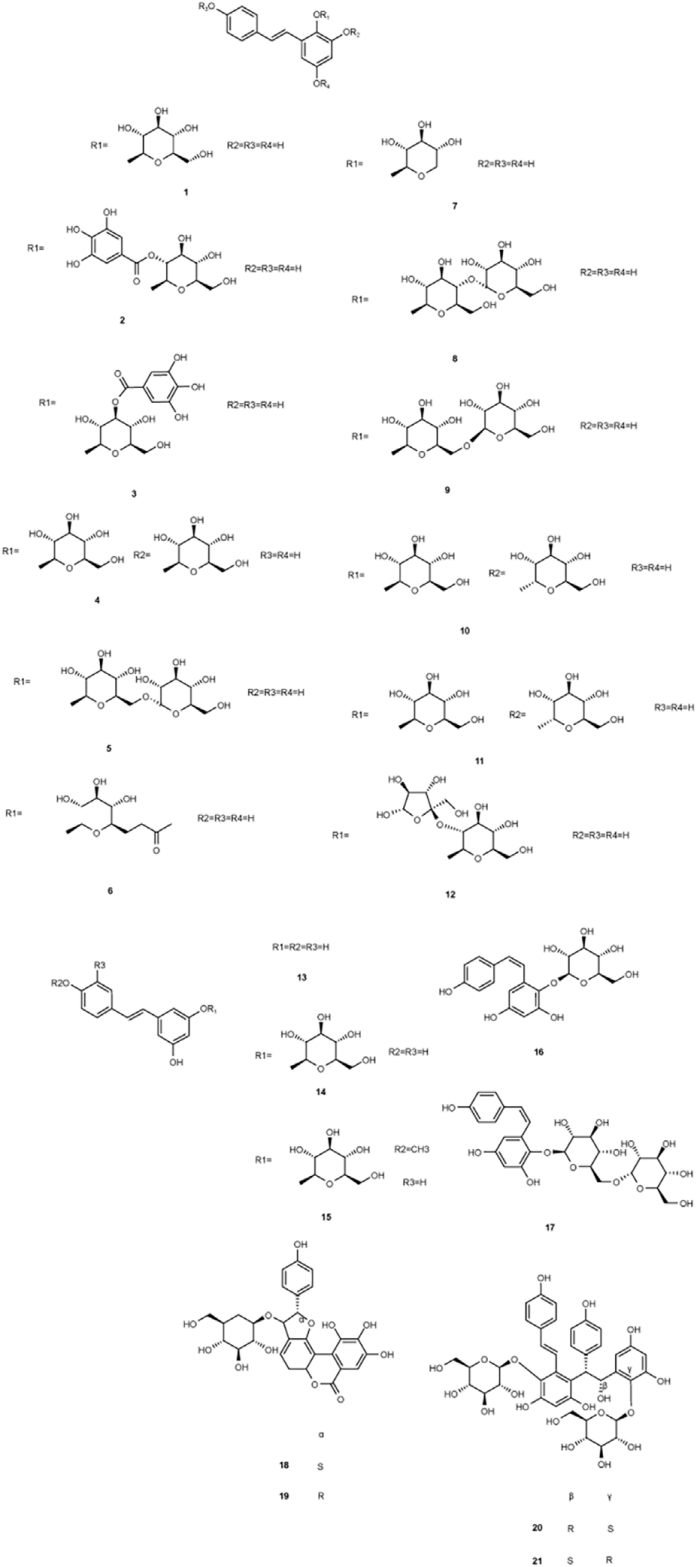
Structures of chemical components 1–21 in *Polygonum multiflorum* Thunb.

**FIGURE 3:**
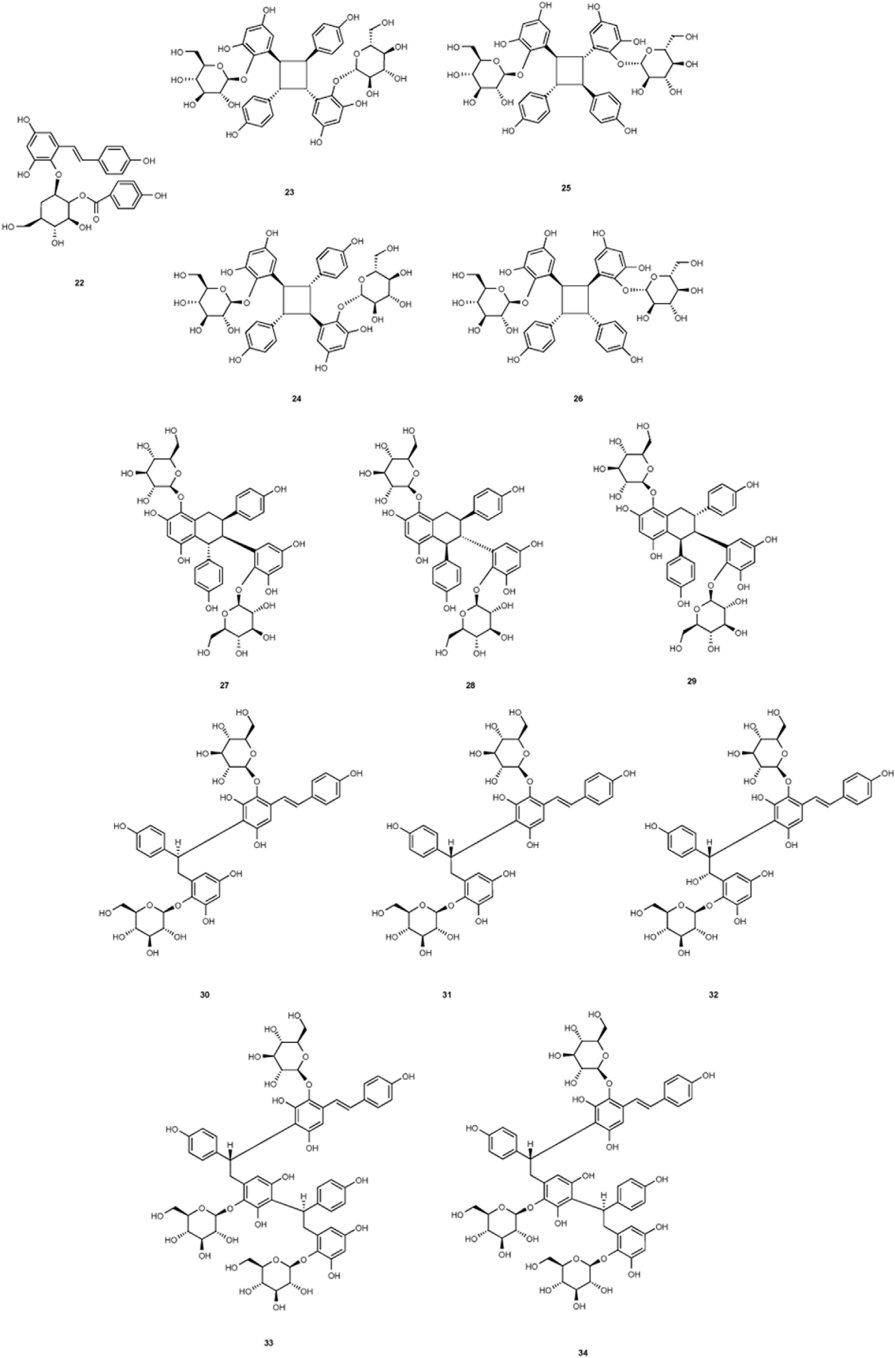
Structures of chemical components 22–34 in *Polygonum multiflorum* Thunb.

**FIGURE 4:**
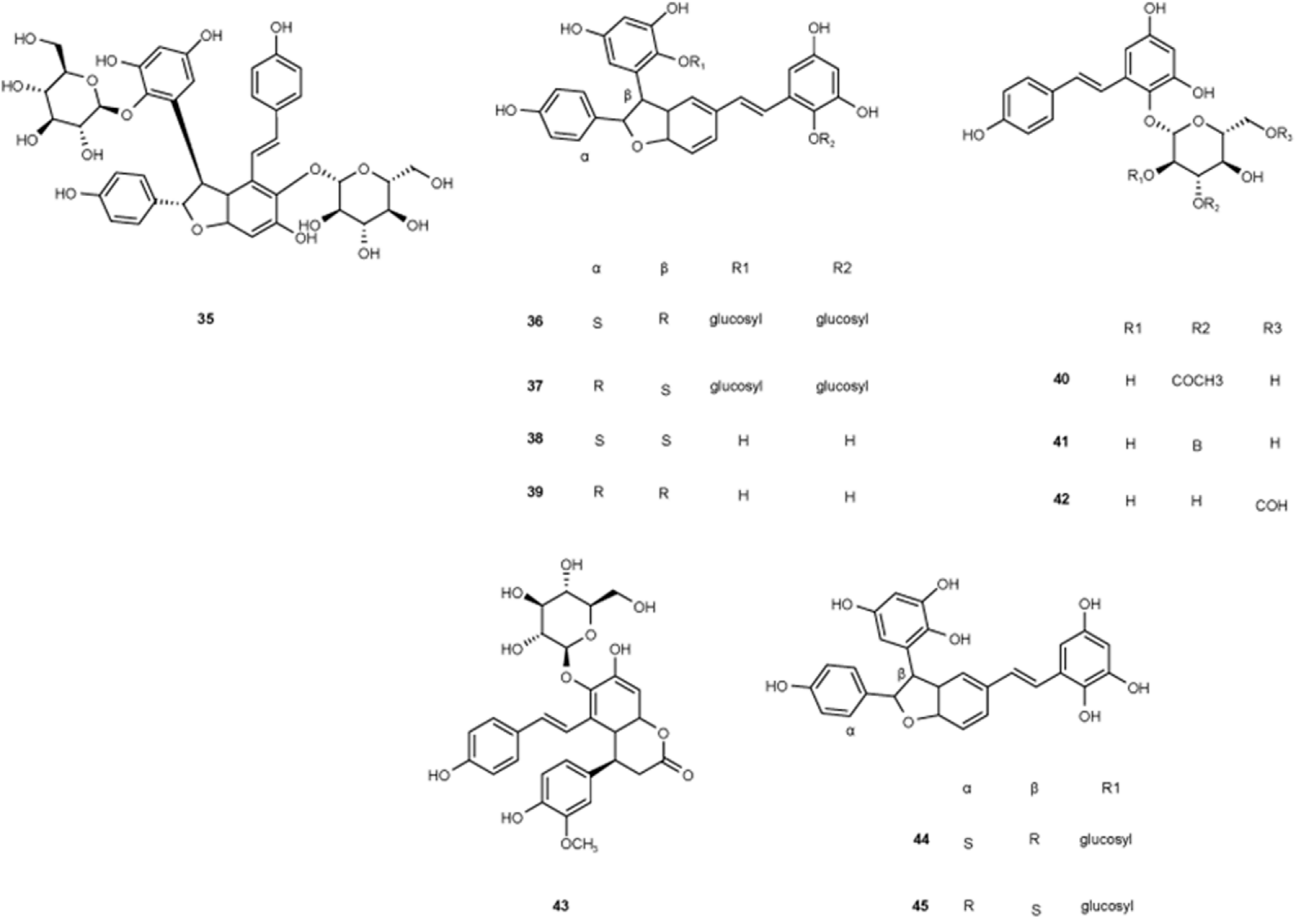
Structures of chemical components 35–43 in *Polygonum multiflorum* Thunb.

The discovery of stilbenes in PM began in 1976 with the characterization of 2,3,5,4′-tetrahydroxystilbene-2-O-β-D-glucopyranoside. Since then, many stilbenes were found, such as 2,3,5,4′-tetrahydroxystilbene-2-O-β-D-glucopyranoside, rhaponticoside, physcion, emodin-8-O-glucoside etc. (Lin et al., 2015) From 2015 to 2020, most of the new compounds isolated are majorly dianthrones and stilbenes (Teka et al., 2021). In 2016, four new dianthrones were isolated by Yang et al., naming polygonumnolides C1-C4 from PM (Yang et al., 2016). After the isolation of polygonumosides A-D in 2014, a new type of stilbene, polygonumoside E was isolated and characterized in 2016 (Yan et al., 2014; Zhao et al., 2016). Zhang and Cui found a new stilbene with HPLC, and named it 2, 3, 5, 4′-tetrahydroxystilbene-2-O-(2″O-p-Phydroxybenzoyl)-β-D-glucoside (Zhang and Cui, 2016). In 2017, a new type of polygonimitin was named as thunberginol-C-6-O-β-D-glucopyranoside. Moreover, polygonumnolide D and polygonumnolide E were found by Yang et al., 2017. The team of Yang continued their research and in 2018, they isolated seven new polygonumnolides, naming polygonumnolide A1, A2, A3, A4, B1, B2 and B3 (Yang J. et al., 2018). In 2018, Li et al. isolated seven new dimeric stilbenes named as multiflorumisides A-G (Li et al., 2018). In the next year, they isolated 5 new oligomeric stilbenes naming multiflorumiside H-L, among which Multiflorumiside H and J showed moderate suppressive effect against nitric oxide production (Li et al., 2019). In 2020, Another research group isolated 9 new stilbenes, named them as polygonibene 1, polygonibene 2, polygonibene 2a, polygonibene 3, polygonibene 3a, and polygonibene 4, 5, 6, and 7 (Nguyen et al., 2020) (Figures 2–4).

#### 3.2.2 Quinones

Quinones, which are abundant in nature, typically possess a basic structural pattern where ortho- or para-substituted diones are conjugated to an benzoquinone or to expanded polycyclic aromatic systems. These compounds exert antioxidant-related pharmacological actions including neuroprotective effects, anti-inflammation, anticancer, hepatoprotective effects and anti-aging, etc. (Zhao and Zheng, 2023) However, quinones can lead to an inhibition of cytochrome P450 enzymes (CYP450 enzymes) and UGTs, resulting in hepatotoxicity.

**FIGURE 5:**
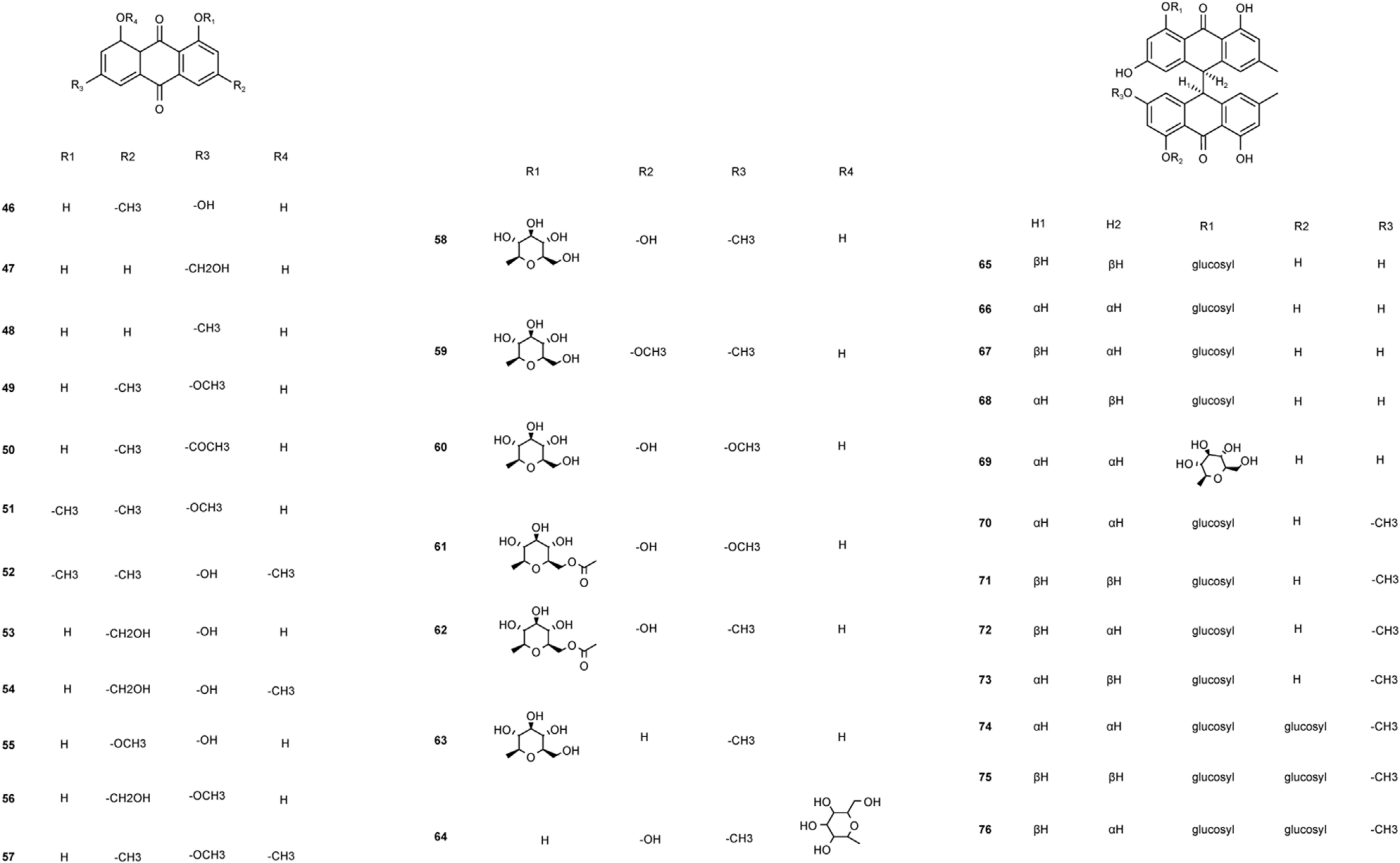
Chemical structures of quinones in *Polygonum multiflorum* Thunb.

The first quinone identified in PM was 2-Methoxy-6-acethyl-7-methyliuglone, isolated in 1993. In subsequent research, Yang et al. isolated seven new polygonumnolides, including cis-emodin dianthrone, in 2018 (Yang et al., 2018). In 2021, Yang et al. first developed the UHPLC-QQQ-MS/MS method to simultaneously determine six dianthrones in PM, including trans-emodin dianthrones, and cis-emodin dianthrones (Yang et al., 2021), marking a significant advancement in analytical techniques (Figure 5).

#### 3.2.3 Other components

In addition to quinones, PM also contains flavonoids and chromenones. Flavonoids, polyphenolic secondary metabolites found in various plants and diets, consist of a structural backbone of 15 carbon atoms and are composed of two benzene rings and heterocycles. These compounds exhibit multiple biological activities, including antioxidant, cardio-protective, hepatocyte-protective, and anti-cancer effects, and are primarily recognized for their liver-protective properties.

**FIGURE 6:**
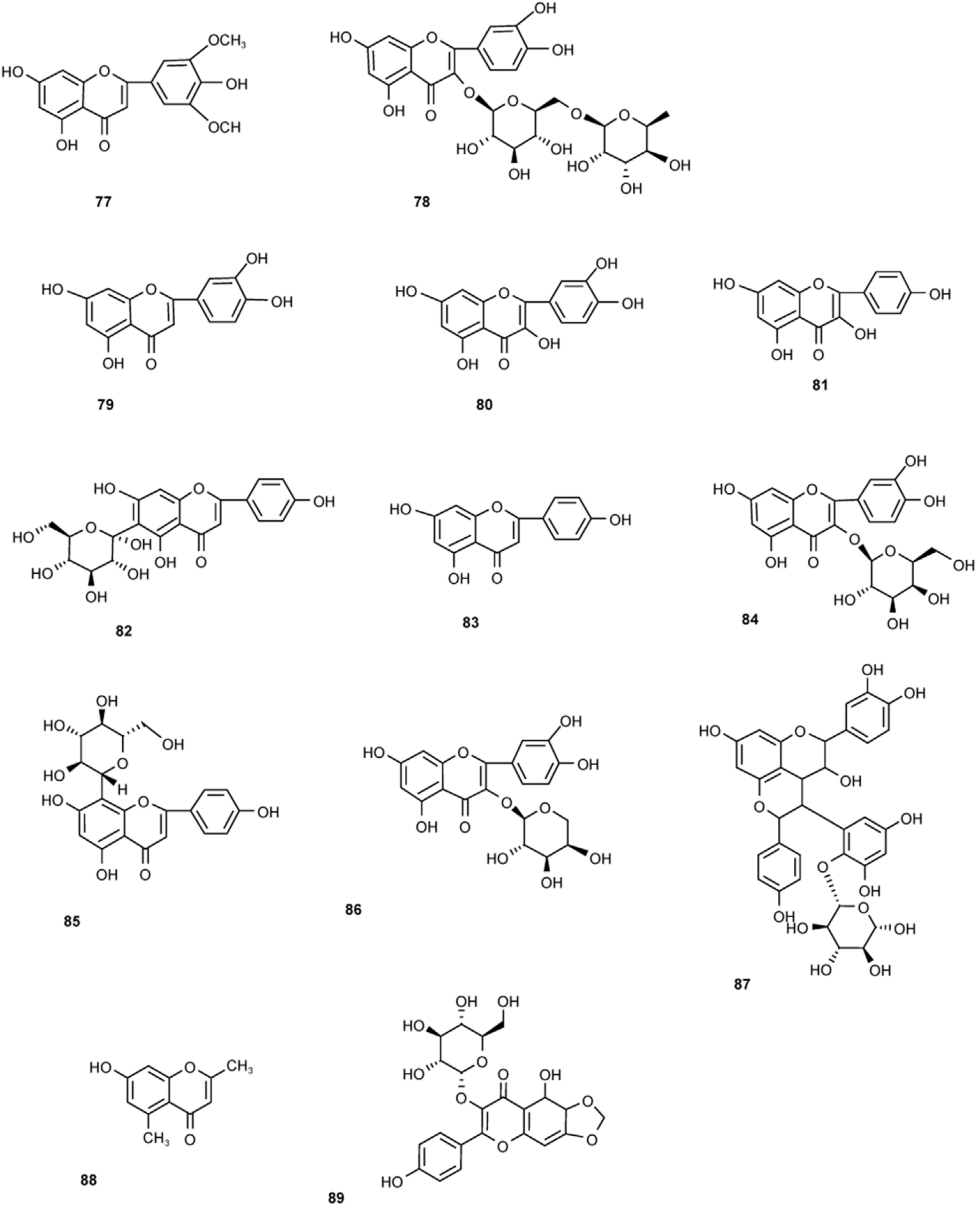
Chemical structures of other components in *Polygonum multiflorum* Thunb.

Two new types of chromenones, 2,5-dimethyl-7-hydroxychromone and 2-(2-hydroxylpropyl)-5-methylchromenone-7-O-β-D-glucopyranoside, and one new type of flavonoid, trycin-7-O-β-D-glucopyranoside, was isolated from PM in 2016 (Yan et al., 2014). With an effect of protecting liver, flavonoids may not be the toxic components in PM (Figure 6).

Although the above components cause liver injury when administrated in large quantities, the quantity of components is very low in the clinical dosage of PM. Therefore, *polygonum multiflorum*-induced liver injury (PM-DILI) may not be independently caused by the above components. The components may play a secondary or synergistic role in the occurrence of PM-DILI (Zhai et al., 2021). Recognition of this may contribute to a more complete understanding of the complex multi-component mechanisms of PM-DILI.

## 4 The toxicity of PM

### 4.1 The toxicity of PM is mainly recognized as liver injury

PM has been recognized as a toxic herb since the toxicity was first reported by Hongkong scholars in 1996. The herb’s toxicity encompasses hepatotoxicity, nephrotoxicity, and embryotoxicity. Hepatotoxicity is linked to stilbenes and quinones, nephrotoxicity and embryotoxicity are mostly linked to stilbenes. With hepatotoxicity being the most significant, it manifests in three forms: intrinsic, idiosyncratic, and indirect toxicity (Xiao et al., 2023).

Intrinsic toxicity refers to the predictable and dose-dependent effects caused by the inherent toxic substances in PM. This type of toxicity, which can be replicated in healthy animal models, is typical of traditional herbs and can often be mitigated through conventional processing methods. Idiosyncratic toxicity targets specific vulnerable populations and is linked to individual immune responses, metabolic processes, and genetic factors. This form of toxicity is less acknowledged in TCM, and its detoxification has not been extensively studied. Indirect toxicity arises from the pharmacological impact of biologically active compounds. It is challenging to replicate in animal models and remains an area of ongoing research (Table 3).

**TABLE 3:** Comparison of intrinsic toxicity and idiosyncratic toxicity.

	Intrinsic toxicity	Idiosyncratic toxicity
cause	Over-dose of usage	Immunity, metabolism and genes of specific patients
population affected	Affects all individuals	Affects only susceptible individuals
relationship with dose used	Dose-dependent	Not dependent on dose
clinically predictable	Predictable and can be avoided	Not predictable
courses after exposure	Predictable courses of disease after exposure	Variable courses of disease after exposure
liver characteristics	Distinctive liver toxicity	Variable liver pathology
animal models	Predictable and can be copied within animal models	Not able to be copied within animal models

#### 4.1.1 Intrinsic toxicity

Intrinsic toxicity, the most prevalent form of PM-DILI, is characterized by a dose-response relationship. This relationship suggests that within certain limits, the dosage of the drug correlates directly with its therapeutic effect, making the prediction and control of intrinsic toxicity more straightforward.

Two primary components of PM, 2,3,5,4′-tetrahydroxystilbene-2-O-β-D-glucoside and emodin (EMD), are chiefly responsible for this toxicity. Intrinsic toxicity is mainly caused by the toxicity of these two components, with a series of characteristics. A study found that different dose concentration of emodin can increase the expression of CYP2E1, 2B6, 1A2, 3A4, 2C9, 2D6, 7A1 mRNA, inducing the expression of CYP1A1 and CYP1B1 in a dose-dependent manner (Wang et al., 2016). And emodin intake can induce the enzymes of CYP1A, CYP2E1, and CYP2B (Wang et al., 2015). Emodin and emodin methyl ether have a strong inhibitory effect on CYP1A2, and a medium inhibitory effect on CYP2C9, CYP2D6 and CYP3A4 (Zheng et al., 2021).

While 2,3,5,4′-tetrahydroxystilbene-2-O-β-D-glucoside alone does not exhibit hepatotoxicity, it exacerbates liver damage when combined with sub-toxic doses of acetaminophen (APAP) in mice. 2,3,5,4′-tetrahydroxystilbene-2-O-β-D-glucoside aggravated hepatic reduced glutathione (GSH) depletion and APAP-cysteine adducts formation induced by APAP in mice (Xu et al., 2017), showing that 2,3,5,4′-tetrahydroxystilbene-2-O-β-D-glucoside may exacerbates the hepatotoxicity caused by APAP. Another study investigated effects of 2,3,5,4′-tetrahydroxystilbene-2-O-β-D-glucoside on the hepatotoxicity caused by DEN. 2,3,5,4′-tetrahydroxystilbene-2-O-β-D-glucoside inhibited liver injury and inflammatory cell infiltration in DEN-treated mice. Besides, 2,3,5,4′-tetrahydroxystilbene-2-O-β-D-glucoside also attenuated DEN-induced accumulation of reactive oxygen species (ROS), pro-inflammatory cytokines, and DNA damage. Both the study indicated that 2,3,5,4′-tetrahydroxystilbene-2-O-β-D-glucoside exacerbates the hepatotoxicity induced by drugs in mice (Yu et al., 2020).

Clinical symptoms of liver toxicity caused by PM are mainly related to digestive system. Loss of appetite and oil aversion are two most emphasized symptoms. Growth of body mass is also reported in many studies. The liver fat growth may be more severe in female than male, indicated in a liver pathological section of rats fed with PM decoction for 3 months (Hu et al., 2009). Scholars found that transaminase and bilirubin (BIL) levels were higher in patients with liver injury caused by PM, and related jaundice is also detected in some patients. All the findings indicated that PM may disturb the metabolism of transaminase and BIL (Tang and Mei, 2017). BIL metabolism is related with UGT of phase II metabolism pathway, and among the isomers of UGT, the core enzyme related to BIL metabolism is UGT1A1 (Chang, 2020). Thus, PM may increase time of BIL metabolism by inhibiting enzyme UGT1A1 in phase II metabolism pathway (Zheng et al., 2021).

#### 4.1.2 Idiosyncratic toxicity

Idiosyncratic drug-induced liver injury (IDILI) affects only a small subset of the population with specific predispositions, and does not exhibit a dose-response relationship, making it clinically unpredictable. Unlike other forms of toxicity, IDILI does not correlate directly with drug dosage, and its underlying pathophysiology remains poorly understood.

The exact pathophysiology of IDILI is multi-factorial involving drug or pharmacological factors (lipophilicity, dose, etc.), environmental factors, and host factors (genetics, immunity, possibly microbiome) (Chen et al., 2013; Stephens et al., 2014). Over the past decade, studies have found that polymorphisms in Human Leukocyte Antigen (HLA) molecules are associated with many drugs that cause IDILI, suggesting a role for the immune system in this pathology (Kaplowitz, 2013). HLA associations clearly point to the involvement of the adaptive immune system in IDILI (Dara et al., 2016).

Epidemiological and clinical data suggest that PM-DILI is an immune-mediated idiosyncratic liver injury. Results showed that 2,3,5,4′-tetrahydroxystilbene-2-O-β-D-glucoside isolated from PM could activate CD4+T and CD8+T in the liver, and could upregulate the levels of inflammatory cytokines including TNF-α, IFN-γ, IL-18, perforin and granzyme B in the liver tissues (Liu et al., 2024). Both PM water extract and cis-stilbene glucoside, the susceptibility component of hepatotoxicity, could cause hepatotoxicity in the mice pre-stimulated with TNF-α (Zhang et al., 2022). Dose of PM caused abnormal liver biochemical indicators and liver tissue damage in MIS model rats (Tu et al., 2019).

Recent attention has focused on IDILI triggered by LPS. Mice pretreated with a modest inflammatory dose of LPS became susceptible to intrinsic liver injury induced by nontoxic doses of APAP (Lai et al., 2018). With normal dose of PM engaged, HE staining of rats’ liver showed mild swelling and local chronic inflammation. The results suggest that PM-DILI induced by LPS is related to inflammation, and this type of hepatotoxicity has no dose-dependence (Pang et al., 2015). Increased expression of cytokines and chemokines, activation of inflammatory cells, depression of PPAR-γ pathway, and stimulation of the TLR-NF-κB pathway, all indicated that the innate immune response may be involved with PM-DILI (Luyendyk et al., 2003; Deng et al., 2006; Zou et al., 2009; Maddox et al., 2010). Several drugs causing IDILI in humans also induce IDILI in animals with a normal dose of LPS, such as diclofenac, sulindac, ranitidine, amiodarone, halothane, and trovafloxacin (Zhu et al., 2019). Immunoinflammation may regulate the susceptibility to IDILI.

Additionally, TCM syndromes may influence PM-DILI susceptibility. After feeding water extract of processed PM, the serum inflammatory mediator TNF-α content of the rats with kidney-yang deficiency significantly increased. Processing of PM has certain liver damage to kidney-yang deficiency model (Bywpgxolgfy, 2016).

### 4.2 Central mechanisms of PM-DILI: Enzyme deficiency and inflammatory response interruption

Enzyme deficiency involves both CYP450 enzymes and UGTs, along with the presence of the HLA-B*35:01 gene in patients. In terms of drug effects, PM-DILI is characterized by disrupted inflammatory responses and impaired metabolism of BIL and bile acids (BA). As for the drugs, interrupting the inflammatory response, BIL and BA metabolism are the main aspects.

#### 4.2.1 Deficiency of metabolism enzymes

**FIGURE 7:**
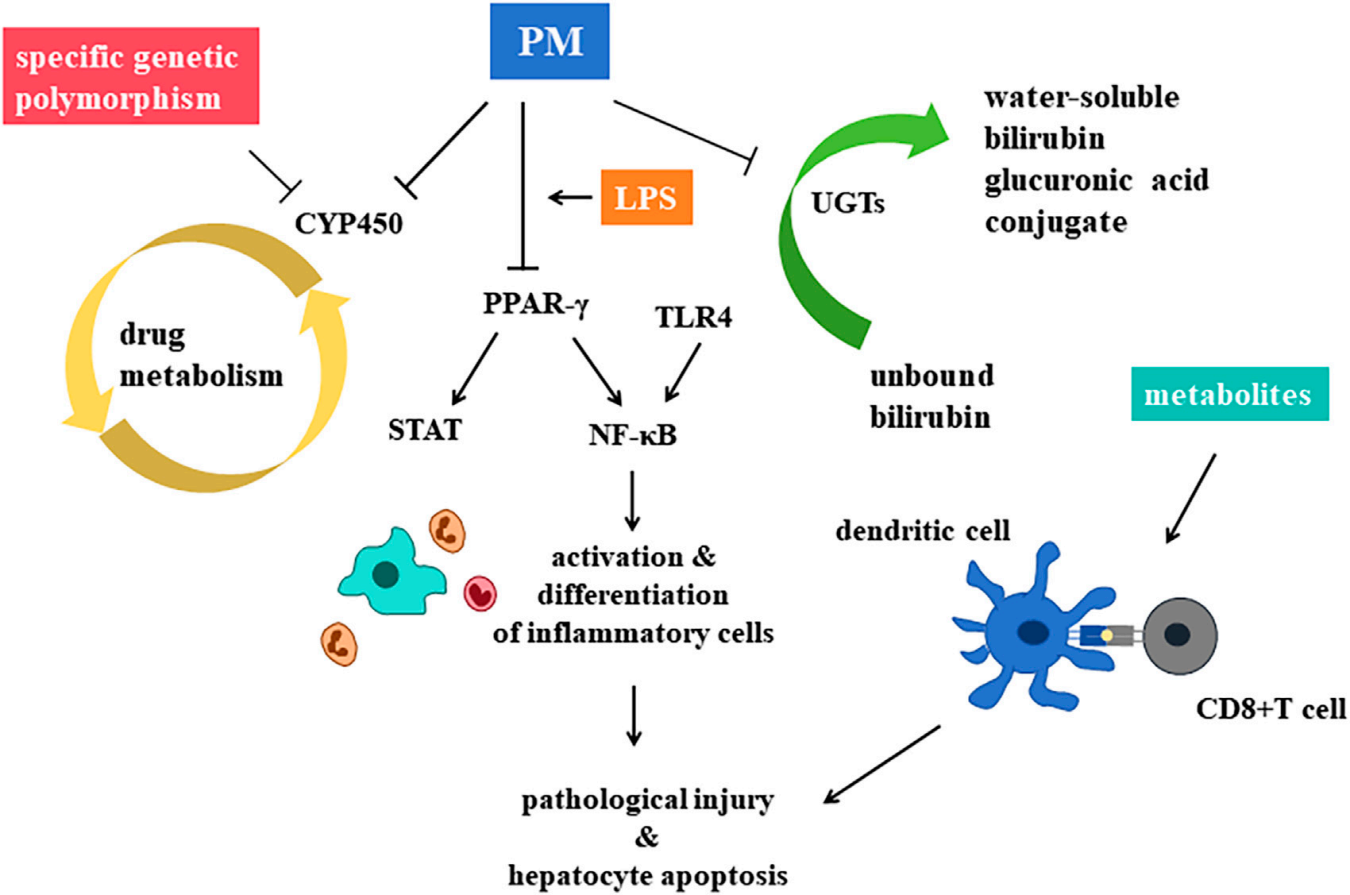
Central mechanisms of hepatotoxicity of *Polygonum multiflorum* Thunb. are recognized as the inhibition on CYP450 enzymes the decrease of PPAR-γ, and the inhibition of UGTs.

Drug metabolism pathway *in vivo* can be divided into phase Ⅰ and phase Ⅱ (Ren et al., 2018). The metabolism of PM is mainly associated with CYP450 enzymes of the phase Ⅰ metabolism, and UGT enzymes of the phase Ⅱ metabolism (Figure 7).

##### 4.2.1.1 Phase Ⅰ metabolism pathway

CYP450 is an important enzyme in drug induced hepatotoxicity, vital for the process of phase Ⅰ metabolism pathway. The main role of CYP450 is to make structural modifications to drug molecules, such as adding a hydroxyl group to the molecular structure to make it more hydrophilic. Many traditional Chinese medicine, PM included, induces or inhibits this enzyme, leading to the accumulation of active metabolic components of drugs, or leading to decreased drug efficacy, increased adverse reactions or toxicity (Mao et al., 2016).

Metabolism of many drugs depends on CYP1A2, such as phenacetin, caffeine, clozapine, tacrine, propranolol and mexiletine, etc. (Hou et al., 2016) PM extractions can significantly decrease the activity and mRNA expression of liver CYP1A2 enzyme (Guo et al., 2021). Another study found that genetic polymorphism of CYP1A2 in patients with PM-DILI is different from that of normal people (Ma et al., 2014). Thus, both the genetic polymorphism of CYP1A2 and the inhibition of PM on CYP1A2, leading to the accumulation of toxic components, may be the mechanism of PM-DILI. Besides, different processing methods of PM can lead to different regulation of mRNA expression levels of drug metabolizing enzymes CYP2E1, CYP3A4 and CYP1A2 (Li et al., 2021). As a result, processing may affect the regulation of CYP450 enzymes, leading to detoxification of PM.

##### 4.2.1.2 Phase Ⅱ metabolism pathway

UGTs is the main metabolic catalytic enzyme in the phase Ⅱ metabolic reaction, metabolizing nearly 35% of drugs and inactivate them. UGTs can transfer the glucuronic acid group of the cofactor uridine diphosphate glucuronic acid (UDPGA) to the hydroxyl group of drug molecules, so as to improve the water solubility of drugs and facilitate their excretion through urine or bile, leading to the occurrence of PM-DILI (Yang et al., 2021).

Glucanosyltransferase 1 family polypeptide A1 (UGT1A1) is a phase Ⅱ metabolic enzyme in the liver. It is related to the catalyze of unbound bilirubin (UCB), it can transform UCB into water-soluble BIL glucuronic acid conjugate, so that UCB can be secreted into bile for elimination. PM has been observed to inhibit UGT1A1, a key enzyme in BIL metabolism, thereby disrupting the conversion of UCB into its water-soluble form and promoting its accumulation, which could lead to hepatotoxicity (Wang Q. et al., 2022).

The toxicity of PM may result from more than one single pathway. Scholars found that the main components of PM such as emodin, rhein, emodin methyl ether, etc. had toxicity on liver cell through inhibiting the activity of CYP1A2 and CYP2C9 and competitively inhibiting the activity of BIL rate-limiting enzyme UGT1A1 (Zheng and Wang, 2019). Besides, there are studies demonstrated the mechanisms such as the induction of apoptosis and the inhibition of liver cell proliferation. Lipid metabolism, bile acid metabolism and energy metabolism are involved. Oxidative phosphorylation pathways is also related according to proteomics (Zhang et al., 2016). Functions of these pathways are still under cultivating (Zhu et al., 2019).

#### 4.2.2 Interruption of inflammatory response

Interruption of the inflammatory response is a key mechanism underlying hepatotoxicity. Peroxisome proliferator activated receptor-γ (PPAR-γ), a member of the nuclear receptor superfamily, plays a critical role in modulating inflammatory and anti-inflammatory signaling pathways. PPAR-γ can inhibit NF-κB and STAT pathways, restraining inflammatory cells, retarding hepatotoxicity.

Based on the model of LPS induced IDILI, He Lanzhi et al. (He et al., 2017) explored the effects of PM’s alcohol extract on hepatotoxicity. They discovered that toxicity might be linked to the over-expression of inflammatory factors and the suppression of the PPAR-γ pathway *in vivo*. PPAR-γ agonists can significantly reduce the levels of ALT and AST in rats plasma, alleviating pathological injury and hepatocyte apoptosis, significantly promote the expression of PPAR-γ and inhibit the expression of NF-κB p65 in liver tissue. Thus, the abnormal decrease of PPAR-γ and the over expression of inflammatory factors may lead to the hepatotoxicity, and PPAR-γ may be the target point of PM-DILI.

Furthermore, activation of the TLR4/IRF-3 signaling pathway has also been implicated in PM-DILI. Unlike dose-dependent mechanisms, hepatotoxicity here is associated with the expression levels of proteins in the TLR4/IRF-3 pathway (Xue et al., 2020).

#### 4.2.3 Genetic risk factor

In the last decade, numerous genome-wide association studies (Kaliyaperumal et al., 2018) have linked human leukocyte antigens (HLAs) to IDILI susceptibility. Scholars found that the HLA‐B*35:01 allele is a genetic risk factor for PM‐DILI and a potential biomarker for predicting PM‐DILI in humans (Lin et al., 2017).

Three models have been put out for the possible mechanisms of T cell-mediated drug hypersensitivity regulated by HLA: the hapten/prohapten model, the pharmacological interaction model and the altered peptide repertoire model (Yun et al., 2012; Uetrecht and Naisbitt, 2013; Redwood et al., 2018). The hapten/prohapten model is the main hypothesis, and is widely studied. Other two hypothesis may act as an alteration to the hapten/prohapten model. As one of the HLA-associated forms of DILI, the mechanism of PM-DILI is associated with the interaction between antigen-presenting cells (APCs) and T cells. After the metabolism of toxic components in the liver, the metabolites can be taken by dendritic cells (DCs), forming adducts covalently with self-proteins, processing in the endoplasmic reticulum, triggering the maturation of DCs. HLA-B*35:01 molecules then present the adducts at the surface of cells, waiting for the activation of CD8+T cells by interacting with T cell receptors (TCRs) (Rodriguez-Pena et al., 2006; Megherbi et al., 2009; Qin et al., 2016; Jiang et al., 2017; Qin et al., 2018).

Besides, FXR gene may also be related to PM-DILI. Scholars screened and verified that the hepatotoxic components aloe emodin-8-O-β-D-glucoside and emodin-8-O-β-D-glucoside in PM could significantly inhibit FXR gene expression through molecular docking and *in vitro* cytotoxicity tests.

In conclusion, gene mechanisms of hepatotoxicity of PM varies a lot, most of the studies emphasize the alterations of drug-metabolizing enzymes as the main mechanism, Immune-Related Gene Polymorphism, BIL-BA metabolism pathway and HLA related T cell-mediated drug hypersensitivity are also important mechanisms.

## 5 Traditional processing and detoxification of PM

**FIGURE 8:**
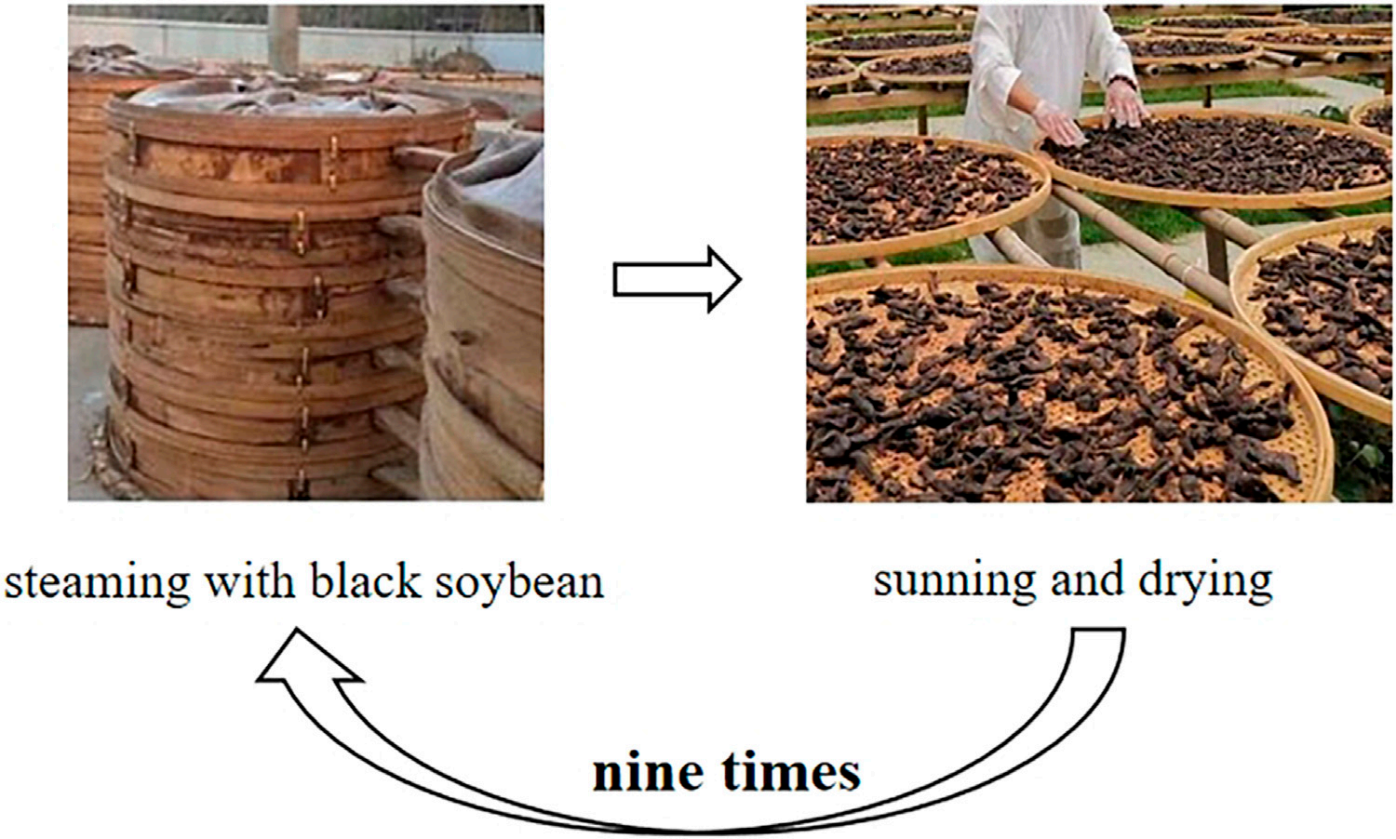
The most widely used processing method: nine times of steaming and sunning.

Traditional Chinese Medicine has utilized various processing methods for decades. There are many processing methods for PM: Wen PE, nine cycles of steaming and sunning, stewing, steaming, etc. Processing is often incorporating with various excipients to modify the herb’s chemical profile and reduce toxicity. After processing, chemical components in PM changed, thus lead to a reduction in toxicity. What’s more, both the methods of processing and excipients added can detoxify PM, as well as increase the effect of it (Figure 8).

### 5.1 Processing detoxification can detoxify PM by changing its chemical components

Processing significant increased content of polysaccharide in PM. Results showed that raw PM and processed PM both comprised Man, Rha, GlcA, GalA, Glc, Ara and Xyl, but markedly differed in polysaccharide yield, molar ratio of monosaccharide composition and Glc/GalA (Wang et al., 2023). The fingerprint of monosaccharide composition also demonstrated that Glc and GalA could be used as differential markers (Gu et al., 2022). It was found that the addition of black beans and steaming times would affect the content and composition of polysaccharide in PM significantly (Fan et al., 2024).

The content of 2,3,5,4′-tetrahydroxystilbene-2-O-β-D-glucoside decreased after processing. Researchers found that during the processing, maillard reaction occurred, producing a large number of compounds, including acetone alcohol, 2, 3-butanediol, succinic acid, 2, 3-dihydro-3, 5-dihydroxy-6-methyl-4H-pyranone (DDMP), 5-hydroxymethyl furfural (5-HMF) and its derivatives. 2,3,5,4′-tetrahydroxystilbene-2-O-β-D-glucoside was converted into a series of derivatives through the esterification reaction with small molecular compounds. Resveratrol was the intermediate product of 2,3,5,4′-tetrahydroxystilbene-2-O-β-D-glucoside, which was hydroxylated to form terahydroxystilbene, and then glycosylated to form 2,3,5,4′-tetrahydroxystilbene-2-O-β-D-glucoside. The representative herbal components trans-2,3,5,4′-tetrahydroxystilbene-2-O-β-D-glucopyranoside and cyanidin-3-O-β-glucoside from each herbal medicine were selected to investigate the processing mechanism at the supramolecular level (Bai et al., 2022). The co-assembly of 2-O-β-D-glucopyranoside and cyanidin-3-O-β-glucoside that leads to supramolecular aggregates discovered here may imply the underlying mechanism of processing PM with black beans (Liu et al., 2023). After nine cycles of steaming, the amount of stilbenes and combined quinones decreased (Zheng et al., 2021).

There are some other components different in raw PM and processed PM. High-performance liquid chromatography studies on different processing methods reveal that stewing PM leads to the hydrolysis of combined quinones into their free forms, thereby diminishing the herb’s toxicity (Ding et al., 2023). Main components of PM such as 2,3,5,4′-tetrahydroxystilbene-2-O-β-D-glucoside, emodin methyl ether and emodin increased significantly (Liu MJPJ. et al., 2020). Five compounds were different in raw and processed PM: α-arabinose, α-galactose, proline, isomer of daidzein and isomer of genistein, which may be potential active ingredients that affect the processing of PM (Chen et al., 2021). Quinones can be decomposed into gallic acid, resulting in an increase in the content after processing. After processing, gallic acid substances in PM increased, and the proportion of quinones decreased. Tannins in PM would be destructed with processing, and may be converted into gallic acid, which further increased the proportion of gallic acid in PM (Ke et al., 2021) (Table 4).

**TABLE 4:** Chemical components before and after processing.

Chemical components	Changes after processing
polysaccharides	The contents of monosaccharides and disaccharides decreased, the total content of carbohydrates increased, and the molecular weight of PM polysaccharides decreased
stilbene glycosides	The overall content of stilbene glycosides decreased, the proportion of trans/cis-stilbene glycosides decreased, and the amount of cis-stilbene glycosides increased
quinones	The content of emodin decreases, emodin methyl ether increases, gallic acid content increases

Wen PE, a unique method of PM processing, was first documented in “Pao Zhi Da Fa,” and it was only passed down in Jianchangbang. The processing method is to take the PM slices, soak them, add black beans, put them in a boiled medicine jar, add warm water, cover them, simmer them for 48 h, take them out, dry them, and sieve them to remove the black bean dregs; Then mix well with yellow wine, wait for exhaustion, steam for 6 h, stop the fire and seal it overnight, take it out, and dry it. Researchers found that the amount of emodin-8-O-β-D-glucopyranoside、torachrysone-8-O-β-D-glucopyranoside、emodin 8-O-(6′-methylmalonyl)-glucopyranoside and physcion-8-O-β-D-monoglucoside decreased in Wen PE, and AST, ALT, LDH, GGT decreased *in vivo*, indicating that Wen processing decreased the hepatotoxicity in PM (Ding et al., 2022).

Processing detoxification can decrease the toxicity of PM with or without excipients. Black soybeans processing is the most widely used. With the excipients of black beans, 12 components changed significantly: Free quinones decreased first and increased as the steaming time extending. Quinones, cis-2,3,5,4′-tetrahydroxystilbene-2-O-β-D-glucoside, polydatin and hypericin increased first and then decreased. Amount of trans-2,3,5,4′-tetrahydroxystilbene-2-O-β-D-glucoside, resveratrol, epicatechin and rutin all decreased (Zhu et al., 2019). Li Yanyi et al. (Li et al., 2023) also found that the content of stilbene glycoside, quinone, flavonoid decreased after processing, while the amount of phenol increased. In a conclusion, steaming with black soybeans can not only decrease the hepatotoxicity, but also increase the medicinal value of PM.

Except for black soybean juice, there are some other excipients that have been investigated. Different processing excipients can lead to different levels of stilbene glycoside and free quinone. Processing of black soybean juice and ginger juice lead to the highest contents of stilbene glycoside and free quinone, the processing of rice swill with black soybean juice reflected the lowest level of stilbene glycoside, and the content of free quinone was the lowest in the procession of black soybean with *Rehmannia glutinosa* DC. juice (Li et al., 2020). Researchers also found that rice swill could be excipient for detoxification (BX, 2017; Liu et al., 2020), jujubes are also standard excipients for detoxification (Liu et al., 2020). The content of stilbene glycoside in boiled PM is the highest, meaning that the three methods of processing can all effectively retain the active ingredients of PM, while rice swill is the best (BX, 2017).

### 5.2 Processing detoxification can detoxify PM as well as increase its efficacy

Repeated processing, particularly through nine cycles of steaming and sunning, significantly alters the chemical composition of PM, thus lead to a decrease in toxicity and an increase in efficacy (Liang et al., 2018).

*In vivo*, results showed that raw PM and processed PM both exerted hepatoprotective effects by upregulating antioxidant enzymes and repressing lipid peroxidation, and that the polysaccharide yield of processed PM was seven-fold higher than that of raw PM, demonstrating that processed PM has better hepatoprotective effects at the same dose of decoction (Wang et al., 2023). Researchers also found that the immunomodulatory activity of processed PM was significantly better than that of raw PM (Gu et al., 2022).

*In vitro*, more cycles of steaming and sunning can lead to a lower serum ALT and AST in rats, due to lower percentage of stilbenes in processed PM (Zheng et al., 2021). The TBIL and DBIL levels decreased gradually with the increase of steaming and sunning times, suggesting that multiple steaming and sunning may result in a protection for the metabolism of transaminase and bilirubin (Gao et al., 2022). Moreover, it has been reported that processing could increase the amount of phenylethyl olefinic glycoside, increase the effects of protecting nerves, improving memory, anti-aging, decreasing blood lipid and so on, thus stewing can not only decrease the toxicity, but can also increase the benefits of PM (Wang et al., 2022).

Mechanisms of processing is still under investigation. Processing PM with black beans could significantly decrease the apoptosis rate of L02 cells, indicating that the detoxification may related to apoptosis (Chen et al., 2021). Huang Chaowen et al. (Huang et al., 2023) explored the mechanism of PM detoxifying, and results showed that processing of PM can decrease the hepatotoxicity by decreasing autophagy, which may provide new interventions for the detoxification.

### 5.3 Compatible detoxification is a convenient way of detoxifying and is traditionally used in clinical practice

Compatible detoxification is one of the traditional Chinese medicine detoxification methods. It means that one kind of Chinese medicine is used together with another or more kinds of Chinese medicine, so as to achieve the purpose of inhibiting or eliminating the toxicity of Chinese medicine. This approach has been shown to alleviate PM-DILI when used with substances such as cistanche polysaccharides, brown algal oligosaccharides, and chito-oligosaccharides (Ren et al., 2021). *Ganoderma lucidum* (Curtis) P. Karst. was used for biological detoxification of tetrahydroxystilbene glucoside-induced idiosyncratic hepatotoxicity of PM (Lin et al., 2021). Scholars have also studied the compatibility of PM with *Poria cocos* (Schw.) Wolf and *Glycyrrhiza uralensis* Fisch., results showed that the compatibility of PM and *Poria cocos* (Schw.) Wolf is better in detoxification (Pang et al., 2015). Compatible detoxification is a convenient way of detoxifying, the two methods of detoxification is usually conducted together in clinical practice, while mechanism of them is worth investigation (Table 5).

**TABLE 5:** Compatibility of PM and compatible effects.

Compatibility of PM	Compatible effects
*poria cocos* (Schw.) Wolf	Decreasing cytotoxicity and the detoxification effect is better than *Glycyrrhiza uralensis* Fisch
*glycyrrhiza uralensis* Fisch	Decreasing cytotoxicity
*ganoderma lucidum* (Curtis) P. Karst	P-hydroxybenzaldehyde was formed, decreasing cell toxicity
cistanche polysaccharides	Improving histopathological changes, reduce liver index and liver function index, inhibit the increase of spleen index, and inhibit TNFα, IL-1β, MCP-1, MIP-1α
brown algal oligosaccharides	Decreasing inflammatory cell infiltration and hepatocyte necrosis, decreasing serum ALT and AST, and decreasing MIP-1α, TNFα, IL-1α
chito-oligosaccharides	Improving histopathological changes, decreasing liver index and liver function index

## 6 Conclusion

Significant advancements have been made in understanding the pharmacology, toxicology, and processing detoxification of PM. Within the aspect of pharmacology, medications of PM have been cultivated since the usage by the ancient Chinese. It is widely used nowadays in the protection from aging, insomnia and hair-losing. Researcher found that PM is mainly consist of stilbenes, quinones and flavonoids. Stilbenes can lead to a dysfunction of UGTs, resulting in IDILI. Quinones can lead to an inhibition of CYP450 enzymes and UGTs, resulting in hepatotoxicity. As a dietary component, flavonoids are thought to have beneficial effects on human health. Their health-promoting properties are associated with antioxidant, anti-inflammatory, and anticancer properties (Serafini et al., 2010; Procházková et al., 2011; Kumar and Pandey, 2013; Gontijo et al., 2017; Dobrzynska et al., 2020). Flavonoids have a function of protecting liver, which means it may be the protecting components of PM. Within the aspect of toxicology, water extract and alcohol extract of raw and prepared PM have been explored, and more than 103 components, including flavonoids, phospholipids, quinones, stilbenes, have been isolated and characterized. Hepatotoxicity is the main aspect of toxicity of PM, intrinsic toxicity and idiosyncratic toxicity can affect the liver function through mechanisms of metabolism enzymes, inflammatory response and genetic risk factors. Within the aspect of processing detoxification, nine cycles of steaming and sunning, and different types of stewing can change the components in PM, resulting in decreased toxicity and enhanced efficiency. Compatible detoxification is also one of the specific ways of detoxification in TCM, with a convenience in usage, it is commonly applied in clinical practice.

In traditional Chinese medicine, PM is widely used for nourishing liver and kidney, tonifying blood, anti-aging and blackening hair. Ancient Chinese use raw PM to treat *postpartum* fever, and process PM with various excipients of Yellow Rice Wine, black soybean juice, swill, and jujube juice, etc. to treat hair losing, forgetfulness, headache, sores and ulcers. Processed PM is decreased in toxicity and is maximum in pharmacologic effects. However, with multi-targets and multi-components, the exact mechanism of processed PM and the pharmacokinetics *in vivo* are still worth investigating. Updating the research paradigm from the current “one target, one drug” mode to a new “network target, multi-components” mode, TCM network pharmacology approach provides a new research paradigm for translating TCM from an experience-based medicine to an evidence-based medicine system, which will accelerate TCM drug discovery, and also improve current drug discovery strategies (Li and Zhang, 2013; Wang et al., 2020; Wang et al., 2022c).

Different aspects of toxicity on PM have been reported, including hepatotoxicity, nephrotoxicity and embryotoxicity, of which hepatotoxicity is the most important. Based on an examination of the literature, we speculate that the possible reasons are as follows. Firstly, the over-dose of usage may lead to intrinsic toxicity, resulting in the binding of drugs and their RM to host proteins, the depletion of antioxidant systems such as GSH, the activation of stress kinases, the release of strong oxidizing groups such as ROS from mitochondrial stress and mitochondrial dysfunction, endoplasmic reticulum stress, as well as the activation of the innate immune system and local inflammatory response of the liver. Secondly, immunity, metabolism and genes of specific patients affects susceptible individuals, leading to idiosyncratic toxicity, resulting in increased expression of cytokines and chemokines, activation of inflammatory cells, depression of PPAR-γ pathway, and stimulation of the TLR-NF-κB pathway.

Indirect liver injury is a kind of drug liver injury caused by the action of the drug rather than by its toxic or idiosyncratic properties, the mechanism of injury is that drugs indirectly cause liver injury by changing the patient’s original liver disease or immune status (Hoofnagle and Björnsson, 2019), which can be caused by immune checkpoint inhibitors (ICIs), glucocorticoids, monoclonal antibodies, etc. At present, there have been no reports of indirect liver injury caused by PM, and it is worth exploring whether PM can cause indirect liver injury.

Therefore, proper dosage, extractions and components of PM, effects as well as toxicity of PM are still need to be verified. Given the diverse toxicological profiles observed, including hepatotoxicity, nephrotoxicity, and embryotoxicity, further research is essential. Mechanisms of traditional processing and compatible detoxification are worth explored. Changes of chemical components before and after processing, as well as changes of chemical components before and after compatibility are need to be deeply researched.
