# Supplementary material for: Phytochemistry, pharmacology, toxicology and detoxification of Polygonum multiflorum Thunb.: a comprehensive review
Source: Front Pharmacol. 2024 Jun 17;15:1427019. doi: 10.3389/fphar.2024.1427019 (PMC11215120; doi:10.3389/fphar.2024.1427019)
Supplement: Supplementary file 1 [file Table1.DOCX]

Supplementary Material

# Supplementary Figures and Tables

Supplement Table 1. Comparison of intrinsic toxicity and idiosyncratic toxicity.

| Traditional pharmacological effects | Source | Name of formula | Compatibility | Usage |
| --- | --- | --- | --- | --- |
| Blackening beard and hair, strong bones and muscles, Nourish essence | Ji Shan Tang Fang | Qi Bao Mei Ran Pill | Polygonum multiflorum Thunb., Black beans, [Wolfiporia extensa Ginns](https://www.cfh.ac.cn/Spdb/548149.sp)., [Achyranthes bidentata Blume](https://www.cfh.ac.cn/Spdb/12156.sp)., Angelica sinensis Diels., [Lycium chinense Mill.](https://www.cfh.ac.cn/Spdb/31741.sp), Cuscuta chinensis Lam., Cullen corylifolium Medik. | PM a catty (rice swill soaked for three or four days, scraped and peeled, Prepare two liters of clean black beans, spread beans together with PM in a casserole wood steamer, and steam until the beans are cooked, drop the beans, dry, change beans and steam again, repeating nine times, dry them in the sun), [Wolfiporia extensa Ginns](https://www.cfh.ac.cn/Spdb/548149.sp). a catty (peeled, levigated, use water to remove the fascia and float ones, smash the sink ones, mix with ten bowls of milk, dry in the sun), [Achyranthes bidentata Blume](https://www.cfh.ac.cn/Spdb/12156.sp). eight taels (Remove seedlings, wine immersion for one day, add into the steamer at the seventh steaming), Angelica sinensis Diels. eight taels (wine immersion, drying in the sun), Cuscuta chinensis Lam. eight taels (wine immersion, smashed, drying in the sun), [Lycium chinense Mill.](https://www.cfh.ac.cn/Spdb/31741.sp) eight taels (wine immersion, drying in the sun), Cullen corylifolium Medik. four taels (smashed, avoid iron device). |
| Treat pain in bone, waist and knee, itching all over | Jing Yan Fang | He Shou Wu Pill | Polygonum multiflorum Thunb., [Achyranthes bidentata Blume](https://www.cfh.ac.cn/Spdb/12156.sp)., Wine | Choose the big Polygonum multiflorum Thunb. for one catty, [Achyranthes bidentata Blume](https://www.cfh.ac.cn/Spdb/12156.sp) one catty, a liter of good wine, soaking for seven nights, dry in the sun. Mashing powder, pilled with honey. |
| Treat long malaria of Yin deficiency, more feeling of hot than cold | Chi Shui Xuan Zhu | He Shou Wu Pill | Polygonum multiflorum Thunb., Soft-shelled turtle blood, Cinnabar | Mash Polygonum multiflorum Thunb. into powder, pilled with soft-shelled turtle blood, covered with cinnabar. |
| Treating deficiency of qi and blood, long malaria | Jing Yue Quan Shu | He Ren Decoction | Polygonum multiflorum Thunb., Angelica sinensis Diels., Panax ginseng C. A. Mey., Pericarpium Citri Reticulatae., [Zingiber officinale Rosc.](https://www.cfh.ac.cn/Spdb/49703.sp), Wine | Polygonum multiflorum Thunb. two liters, Angelica sinensis Diels. two liters, Panax ginseng C. A. Mey. three liters, Pericarpium Citri Reticulatae. two liters, [Zingiber officinale Rosc.](https://www.cfh.ac.cn/Spdb/49703.sp) three slices, water two bells, make into decoction, drink before malaria. |
| Treating sores and itching | Wai Ke Jing Yao | He Shou Wu Powder | Polygonum multiflorum Thunb., Sophora flavescens Aiton, Saposhnikovia divaricata (Turcz.) Schischk., Mentha canadensis L., Wine | Make Saposhnikovia divaricata (Turcz.) Schischk., Sophora flavescens Aiton, Polygonum multiflorum Thunb. and Mentha canadensis L. into powder, decoct with water as usage, wash the skin with decoction. |
| Treating Scrofula, chronic pharyngitis | Sheng Hui Fang | He Shou Wu Pill | Polygonum multiflorum Thunb., [Ecklonia kurome Okamura](https://www.cfh.ac.cn/Spdb/538578.sp), Moschus, Gleditsia sinensis Lam., Nepeta cataria L., Mutton | Polygonum multiflorum Thunb. two taels, [Ecklonia kurome Okamura](https://www.cfh.ac.cn/Spdb/538578.sp) two taels, Moschus one tael, Gleditsia sinensis Lam. ten pieces. Make them into powder, and pilled them together with mutton, take the pills with Nepeta cataria L. decoction. |
| Treating Scrofula, weakness | Ben Cao Hui Yan | He Shou Wu Pill | Polygonum multiflorum Thunb., Prunella vulgaris L., Bolbostemmatis Rhizoma, Angelica sinensis Diels., [Cyperus rotundus Linn.](https://www.cfh.ac.cn/Spdb/45718.sp), [Conioselinum anthriscoides](https://www.cfh.ac.cn/Spdb/105643.sp) | Polygonum multiflorum Thunb. one catty, peeling, add Prunella vulgaris L. four taels, Bolbostemmatis Rhizoma, Angelica sinensis Diels., [Cyperus rotundus Linn.](https://www.cfh.ac.cn/Spdb/45718.sp) three taels each, and [Conioselinum anthriscoides](https://www.cfh.ac.cn/Spdb/105643.sp) one tael. Pilled with honey. |
| Treating mange all over | Bo Ji Fang | He Shou Wu Powder | Polygonum multiflorum Thunb., Artemisia argyi H. Lév. & Vaniot | Make Polygonum multiflorum Thunb. and Artemisia argyi H. Lév. & Vaniot into powder. Spread on the skin. |
| Treating wind of the large intestine, diarrhea with blood | Sheng Hui Fang | He Shou Wu Powder | Polygonum multiflorum Thunb. | Polygonum multiflorum Thunb. two taels, make into powder, eat before meals. |
| Treating autohidrosis | Bing Hu Ji Jian Fang | He Shou Wu Paste | Polygonum multiflorum Thunb. | Paste Polygonum multiflorum Thunb. with water, and lay it on the navel. |
| Treating tetanus with blood | Wei Sheng Za Xing | He Shou Wu Powder | Polygonum multiflorum Thunb. | Spread the Polygonum multiflorum Thunb. powder on the skin. |
| Treating women blood wind, weakness of limbs, limb paralysis and skin itching, hemorrhoids | Pu Ji Fang | He Shou Wu Pill | Polygonum multiflorum Thunb., Paeonia lactiflora Pall. | Polygonum multiflorum Thunb. one catty, Paeonia lactiflora Pall. two taels, make them into powder, and pilled with paste. |
| Treating Scrofula | Dou Men Fang | He Shou Wu Pill | Polygonum multiflorum Thunb. | Chewing the raw Polygonum multiflorum Thunb. |
| Treating Scrofula, toxic ulcer | Xin Yi Ji | He Shou Wu Paste | Polygonum multiflorum Thunb., Angelica sinensis Diels., Smilax glabra Roxb., Lonicera japonica Thunb. | Polygonum multiflorum Thunb. three catty, Smilax glabra Roxb. eight catty, Angelica sinensis Diels. one catty and eight taels, Lonicera japonica Thunb. one catty, make them into paste like substance. |
